# Detection and Molecular Characterization of Enteric Viruses in Bivalve Mollusks Collected in Arraial do Cabo, Rio de Janeiro, Brazil

**DOI:** 10.3390/v14112359

**Published:** 2022-10-26

**Authors:** Lilian Gonçalves do Nascimento, Sylvia Kahwage Sarmento, Raphael Leonardo, Meylin Bautista Gutierrez, Fábio Correia Malta, Jaqueline Mendes de Oliveira, Caroline Rezende Guerra, Ricardo Coutinho, Marize Pereira Miagostovich, Tulio Machado Fumian

**Affiliations:** 1 Laboratory of Comparative and Environmental Virology, Oswaldo Cruz Institute, Fiocruz, Rio de Janeiro 21040-360, RJ, Brazil; 2 Laboratory of Viral Morphology and Morphogenesis, Oswaldo Cruz Institute, Fiocruz, Rio de Janeiro 21040-360, RJ, Brazil; 3 Laboratory of Technological Development in Virology, Oswaldo Cruz Institute, Fiocruz, Rio de Janeiro 21040-360, RJ, Brazil; 4 Laboratory of Marine Genetics, Department of Marine Biotechnology, Sea Studies Institute Admiral Paulo Moreira (IEAPM), Arraial do Cabo 28930-000, RJ, Brazil

**Keywords:** enteric viruses, bivalve mollusks, molecular methods, norovirus, rotavirus A, hepatitis A virus, human adenovirus, human bocavirus

## Abstract

Viral bivalve contamination is a recognized food safety hazard. Therefore, this study investigated the detection rates, seasonality, quantification, and genetic diversity of enteric viruses in bivalve samples (mussels and oysters). We collected 97 shellfish samples between March 2018 and February 2020. The screening of samples by qPCR or RT-qPCR revealed the detection of norovirus (42.3%), rotavirus A (RVA; 16.5%), human adenovirus (HAdV; 24.7%), and human bocavirus (HBoV; 13.4%). There was no detection of hepatitis A virus. In total, 58.8% of shellfish samples tested positive for one or more viruses, with 42.1% of positive samples contaminated with two or more viruses. Norovirus showed the highest median viral load (3.3 × 10^6^ GC/g), followed by HAdV (median of 3.5 × 10^4^ GC/g), RVA (median of 1.5 × 10^3^ GC/g), and HBoV (median of 1.3 × 10^3^ GC/g). Phylogenetic analysis revealed that norovirus strains belonged to genotype GII.12[P16], RVA to genotype I2, HAdV to types -C2, -C5, and -F40, and HBoV to genotypes -1 and -2. Our results demonstrate the viral contamination of bivalves, emphasizing the need for virological monitoring programs to ensure the quality and safety of shellfish for human consumption and as a valuable surveillance tool to monitor emerging viruses and novel variants.

## 1. Introduction

Foodborne diseases are currently a major public health problem and are associated with a significant economic burden worldwide. It is estimated that the consumption of contaminated food is responsible for 600 million cases of foodborne illness and approximately 420,000 deaths [1]. Enteric viruses are excreted at high concentrations in the stool of infected individuals, which are routinely discharged into the environment [2,3]. High concentrations of enteric viruses can be found in municipal sewage, even after sewage secondary treatment methods, which are only partially efficient in eliminating viral agents [4,5,6]. In this manner, a large concentration of viruses of animal or human origin are widely dispersed in the environment. Thus, the combination of high viral concentrations in environmental waters, the low infective dose required for infection, and their capacity to remain stable and persist in the aquatic environment may result in poor health outcomes if contaminated food or water is consumed [3,7,8,9,10,11].

In recent years, reports of foodborne illnesses of viral origin have increased significantly, and bivalve mollusks are one of the main food groups implicated in outbreaks of acute gastroenteritis (AGE) or hepatitis [12,13]. Norovirus and hepatitis A (HAV) are the most common associated viruses; however, other viral agents, including rotavirus A (RVA), human adenovirus (HAdV), human bocavirus (HBoV), enterovirus, sapovirus, and astrovirus have been detected and/or implicated in foodborne diseases [14,15,16,17,18,19,20,21,22]. Aquaculture production of bivalve mollusks is mainly performed in open seawater systems, where they are exposed to any pollutant present in the seawater (enteric viruses, bacteria and microplastics) during their growth [23,24,25]. These filter-feeder animals are capable of bioaccumulate microorganisms in their tissues at higher concentrations than the surrounding seawater. Therefore, the cultivation and production of shellfish in coastal zones or marine estuaries contaminated by sewage discharge, in addition to the ingestion of raw or undercooked shellfish, represent an important source for contamination and disease transmission [8,10,11,22]. More recently, it was suggested the use of viral detection in bivalves as sentinels to monitor the contamination of SARS-CoV-2 in French coasts [26], and studies from Spain and Italy have reported the detection of SARS-CoV-2 RNA in bivalves samples [27,28].

RVA and norovirus are recognized as leading global causes of AGE requiring hospitalization and outbreaks [29,30]. Noroviruses are single-stranded, positive-sense RNA viruses that belong to the family Caliciviridae and genus Norovirus, classified into 10 genogroups and 49 genotypes that infect a broad range of mammalian host species [31]. RVA, belonging to the genus Rotavirus within the family Sedoreoviridae, are triple-layered, non-enveloped viral particles that comprise eleven segments of double-stranded RNA genome [32,33]. Currently, based on the two capsid proteins, 58 P and 42 G genotypes are recognized to infect humans and animals (https://rega.kuleuven.be/cev/viralmetagenomics/virus-classification/rcwg, accessed on 12 July 2022). HAV is a non-enveloped, single-stranded RNA virus belonging to the genus Hepatovirus of the family Picornaviridae. HAV is the leading cause of viral hepatitis globally, and its incidence varies according to each country’s socioeconomic conditions [34]. The virus is transmitted through the fecal-oral route, mainly by the consumption of contaminated food and water, and is responsible for a considerable number of acute self-limited hepatitis cases worldwide, with seafood constituting one of the main sources of infection, as indicated by several reports [12,13,35].

HAdVs are large (90 nm) non-enveloped, double-stranded DNA viruses that are members of the Adenoviridae family (genus Mastadenovirus) [36]. Currently, they are divided into seven species (A–G) and over a hundred HAdV types were characterized (http://hadvwg.gmu.edu/, accessed on 29 July 2022). HAdV are responsible for a broad spectrum of clinical illnesses, which are typically mild and self-limiting, and are most frequently associated with respiratory infections, conjunctivitis and AGE [37]. Due to their year-round prevalence, environmental stability, and host specificity the HAdVs have been considered an ideal viral marker of human fecal contamination [2,38,39]. First described in 2005, HBoV are small (20 nm) non-enveloped, linear single-stranded DNA virus, belonging to the Bocaparvovirus genus of the Parvoviridae family [40]. These emerging viruses are distributed globally and classified into four genotypes (HBoV-1 to -4). Their epidemiology has been described in several nations worldwide, and they are especially important in pediatric patients with respiratory and gastrointestinal infections [41,42,43,44,45,46,47].

In Brazil, there is limited information on enteric viruses’ contamination of bivalve mollusks. Therefore, our study aimed to determine the detection rates and concentration of enteric viruses (norovirus, RVA, HAV, HAdV and HBoV) in bivalve mollusks samples collected in Arraial do Cabo, Rio de Janeiro, Brazil, using RT-qPCR or qPCR. In addition, viruses detected were molecularly characterized to investigate their genetic variability.

## 2. Materials and Methods

### 2.1. Site Description and Shellfish Sampling

Oysters (*Pseudochama cristella*) and mussels (*Perna perna*) were collected at three areas in the Extractive Marine Reserve of Arraial do Cabo, Rio de Janeiro, Southeastern Brazil, as previously described by Sarmento et al. [17]. A total of 97 bivalve samples (73 mussels and 24 oysters samples) were collected between March 2018 and February 2020. Throughout the study period, samples were collected monthly, with the exception of the period from December 2018 to February 2019, when samples were collected bimonthly. A sample consisted of 12−15 individuals (oysters or mussels) collected at the same time and area.

### 2.2. Viral Recovery Method

Bivalve mollusk samples were transported to the laboratory at 4 °C, where they were immediately processed or stored at −80 °C. The digestive tissues of oysters and mussels samples were processed according to the method described in the revised ISO 15216-1:2017 [48]. Briefly, 2 mL of proteinase K solution (100 μg/mL, Invitrogen, Carlsbad, CA, USA) was added and mixed with each sample’s homogenized digestive tissues (2.0 ± 0.2 g). The samples were vortexed for 5 min and incubated at 37 °C with shaking (320 rpm) for 60 min, followed by 15 min at 60 °C. After incubation, samples were centrifuged at 3000× *g* for 5 min, and the soluble homogenate (~2.5 mL) was collected and stored at −80 °C until nucleic acid extraction. All samples were spiked with 10 µL of the PP7 bacteriophage [6.8 log10 genome copies (gc) μL^−1^], which was used as an internal process control.

### 2.3. Nucleic Acid Extraction

Viral nucleic acids were extracted from 500 μL of homogenate using the NucleoMag^®^ RNA Virus Extraction Kit (Macherey-Nagel, Dueren, Germany) according to the manufacturer’s instructions. Nucleic acids were eluted in 100 µL of elution buffer and either immediately analyzed or stored at −80 °C until use. Each extraction batch included a negative control (sterile water) and in-house positive control (positive stool sample) for each virus tested.

### 2.4. Viral Detection

Norovirus (GI and GII), RVA, HAV, HAdV and HBoV were detected and quantified using a TaqMan^®^-based RT-qPCR or qPCR protocols under the same reactions conditions as previously described (Table 1). Molecular reactions were performed on the Applied Biosystems^®^ 7500 Real-Time PCR System (Applied Biosystems, Foster City, CA, USA) using the SuperScript^™^ III Platinum^™^ One-Step qRT-PCR Kit (ThermoFisher Scientific, Invitrogen Division, Carlsbad, CA, USA) for norovirus, RVA and HAV or TaqMan Universal Master Mix kit (Applied Biosystems, Foster City, CA, USA) for HAdV and HBoV. The set of primers and probes, targeted regions and references are detailed in Table 1. The PP7 bacteriophage, used as an internal process control, was recovered using primers and a probe described by Rajal et al. [49].

For each tested virus, samples that crossed the threshold line showing a characteristic sigmoid curve with a cycle threshold (Ct) value ≤ 40 were regarded as positive. All runs included negative, positive and non-template controls. Viral concentrations, expressed as genome copies per gram of tissue (GC/g), were estimated by using 10-fold serial dilutions [10^6^–10^1^ genome copies (GC) per reaction] of a double-stranded DNA fragment containing the amplification region sequence of each virus (gBlock Gene Fragment, Integrated DNA Technologies, Coralville, IA, USA). Enteric viruses’ calibration curves were as follows: for norovirus GI, y = −3.459 x + 41.83, amplification efficiency (Eff.) = 94.566%, and correlation coefficient (R^2^) = 0.994; for norovirus GII, y = −3.464 x + 40.818, Eff. = 94.379%, R^2^ = 0.974; for RVA, y = −3.474 x + 35.715, Eff. = 94.03%, R^2^ = 0.999; for HAdV, y = −3.408 x + 42.636, Eff. = 96.51%, R^2^ = 0.998; for HBoV y = −3.356 x + 38.304, Eff. = 98.589%, R^2^ = 0.996. To minimize and evaluate inhibitor interference, undiluted and 1:10 diluted nucleic acid samples were tested in duplicate.

### 2.5. Molecular Characterization and Phylogenetic Analysis

Positive samples were subjected to conventional PCR or RT-PCR for genotyping using previously described primers for each virus (Table 1). The generated amplicons of norovirus GII (557 base pairs (bp)), RVA (379 bp), HAdV (301 bp), and HBoV (576 bp) were purified using the QIAquick Gel Extraction Kit (QIAGEN, Valencia, CA, USA) following the manufacturer’s instructions. Purified amplicons were directly sequenced at the FIOCRUZ Institutional Sequencing Platform (PDTIS) with the BigDye^™^ Terminator v. 3.1 Cycle Sequencing Kit (Applied Biosystems, Foster City, CA, USA) on an ABI Prism 3730*xl* genetic analyzer (Applied Biosystems, Foster City, CA, USA). After nucleotide (nt) alignment and edition with Geneious prime 2021.1.1 software (Biomatters Ltd., Auckland, New Zealand), generated consensus sequences with identified genotypes were confirmed in terms of closest homology sequence using the Basic Local Alignment Search Tool (BLAST) server (https://blast.ncbi.nlm.nih.gov/Blast.cgi, accessed on 28 July 2022). Phylogenetic trees were constructed using the maximum likelihood method (2000 bootstrap replications for branch support) in MEGA X v. 10.1.7 [58], with reference sequences obtained from the GenBank database. Nucleotide sequences obtained in this study were deposited in GenBank under the accession numbers: ON815271-ON815277; ON855070-ON855076; ON866925-ON866928 and OP374150-OP374153.

### 2.6. Data Analysis

Statistical analyses were performed using GraphPad Prism version 9.0.0 (GraphPad Software, San Diego, CA, USA). Box-and-whisker plots were produced to illustrate the differences between medians. Viral concentrations (DNA or RNA copy number) recovered in bivalve samples were analyzed for significant differences using the Independent-Samples Mann–Whitney U Test. Enteric virus detection frequencies from different sites and seasons were compared through the Chi-square or Fisher’s exact test. For all analyses, *p* < 0.05 was considered statistically significant.

## 3. Results

### 3.1. Enteric Viruses Single and Co-Detections in Bivalve Shellfish

Over the 24-month study period, we tested a total of 97 bivalve shellfish samples (24 oysters and 73 mussels) collected from three sampling sites in Arraial do Cabo city. Each shellfish sample was tested for the presence of RVA, HAV, HAdV and HBoV. Concerning norovirus detection, our group has published the results for the first 16-month period [17], and the present study includes original results from 20 additional bivalve samples collected between August 2019 and February 2020, which were not included in the previous study. In order to evaluate total norovirus single, co-detection rates, and viral load, we included norovirus data from the initial 16-month period when needed. 

**Figure 1:**
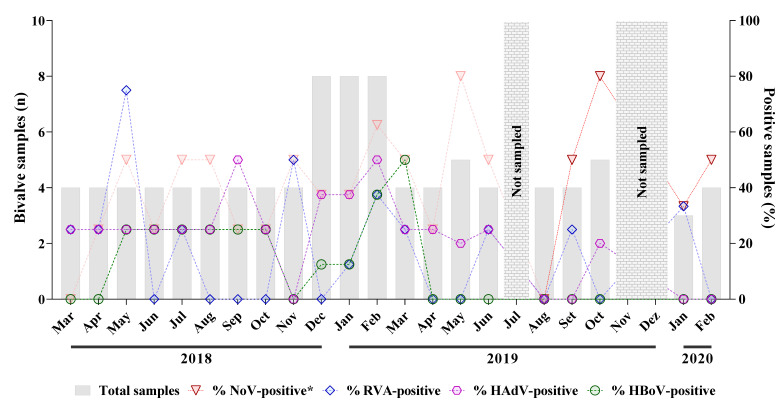
Distribution of bivalve shellfish samples collected from March 2018 to February 2020, with monthly positivity rates for norovirus (NoV), rotavirus A (RVA), human adenovirus (HAdV), and human bocavirus (HBoV) each month from March 2018 to February 2020. Initial norovirus results, published by Sarmento et al. [17], are shown in fading red.

Except for August 2019, at least one sample tested positive each month for one of the enteric viruses investigated. Regarding detection frequency, monthly rates varied considerably among viruses. During the 24-month period, norovirus was the most frequently detected, identified in 42.3% of samples (*n* = 41). In addition, between August 2019 and February 2020, 9 of the 20 collected samples were positive for norovirus, with monthly detection rates ranging from 33.3% to 80%. HAdV was the second most frequently detected virus, with a positivity rate of 24.7% (*n* = 24). HAdV monthly detection rates ranged from 25% to 50%, with higher detection rates in September 2018 and February 2019. RVA and HBoV were detected less frequently, with detection rates of 16.5% (*n* = 16) and 13.4% (*n* = 13) of tested samples, respectively. HAV was not detected in any of the 97 tested samples. The internal process control (PP7 bacteriophage) was detected in 100% of seeded samples, with a recovery rate of 23.4% ± 9.3 [mean ± standard deviation (sd)]. The viral load values of PP7 obtained for all samples ranged from 3.2 × 10^4^ to 5 × 10^6^ GC/g for oysters and 6 × 10^4^ to 1 × 10^7^ GC/g for mussels and the median values were 1.1 × 10^5^ and 7 × 10^5^ GC, respectively. Figure 1 provides a detailed analysis of the monthly positivity rate for each virus.

We also analyzed the frequency of enteric virus distribution by season and sampling site. Norovirus and HAdV were more frequently detected in summer months, whereas RVA and HBoV were detected more frequently during autumn and spring seasons, respectively. However, none of the viruses displayed a seasonal pattern (*p* > 0.05). As for sampling sites, norovirus, HAdV and HBoV detection rates were higher in samples from Farol beach than in samples from Anjos and Forno beaches. Additionally, the majority of co-detections were observed in Farol beach samples. RVA was detected more frequently in samples from Forno Beach.

Regarding single and multiple detections, 58.8% (57/97) of tested samples contained one or more viruses, with 57.9% (33/57) of positive samples testing positive for a single viral agent, 21.1% (12/57) for two viral agents, and 19.3% (11/57) for three viral agents. Additionally, a single oyster sample was contaminated with the four enteric viruses (Table 2).

### 3.2. Enteric Viruses Quantification

Norovirus showed the highest viral load compared to the other enteric viruses. In samples collected between August 2019 and February 2020, only norovirus GII was detected, with an estimated viral load ranging from 4.1 × 10^2^ to 1.2 × 10^7^ GC/g (median of 3.3 × 10^6^ GC/g). In addition, eight of the nine norovirus-positive samples showed Ct values below 26.3 (Ct median of 22). For RVA-positive samples, Ct values varied from 32.2 to 39.9, and RNA concentrations ranged from 8.6 × 10^2^ to 1.6 × 10^5^ GC/g (median of 1.5 × 10^3^ GC/g).

**Figure 2:**
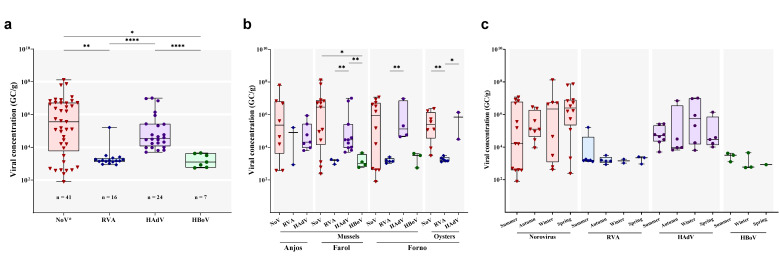
(**a**) Overall variation of enteric viruses’ concentration in bivalve samples and the viral load of enteric viruses by (**b**) sampling site and (**c**) season. Box and whisker plots show the first and third quartiles (equivalent to the 5th and 95th percentiles), the median (horizontal line in the box) and range concentrations [genome copies per gram (GC/g) of digestive tissue]. * *p* ≤ 0.05; ** *p* ≤ 0.01; **** *p* ≤ 0.0001.

Regarding DNA virus quantification, HAdV-positive samples showed viral loads ranging from 5 × 10^3^ to 1 × 10^7^ GC/g (median of 3.5 × 10^4^ GC/g), with Ct values ranging from 26.1 to 37.2, the second highest median concentration detected after norovirus. HBoV DNA concentration ranged from 5.5 × 10^2^ to 4.5 × 10^3^ GC/g, with a median of 1.3 × 10^3^ GC/g, the lowest among the enteric viruses detected. Comparing the viral concentration of detected enteric viruses during the entire study period, we observed a statistically significant difference in norovirus viral load compared to RVA (*p* = 0.0014) and HBoV (*p* = 0.0126) viral loads. Likewise, HAdV viral loads were significantly higher than RVA and HBoV (*p* < 0.0001 for both) (Figure 2a).

Additionally, we analyzed the viral concentration of each virus by sampling site (Figure 2b) and season (Figure 2c). There was no statistical difference comparing the viral loads of each virus among the three sampling sites and different seasons. Analyzing the viral loads for each sampling site separately, we observed a statistically significant difference between norovirus and HBoV (*p* = 0.0445) and among HAdV compared to RVA and HBoV (*p* = 0.0055 and *p* = 0.0015, respectively) in samples obtained from Farol beach. There was also a significant difference in the viral load of samples collected from Forno beach between RVA and HAdV (*p* = 0.0159) in mussels samples, as well as among RVA compared to norovirus and HAdV (*p* = 0.0013 and *p* = 0.0357, respectively) in oysters samples. There was no difference in viral load among the viruses detected in samples from Anjos beach.

### 3.3. Enteric Viruses Characterization 

**Figure 3:**
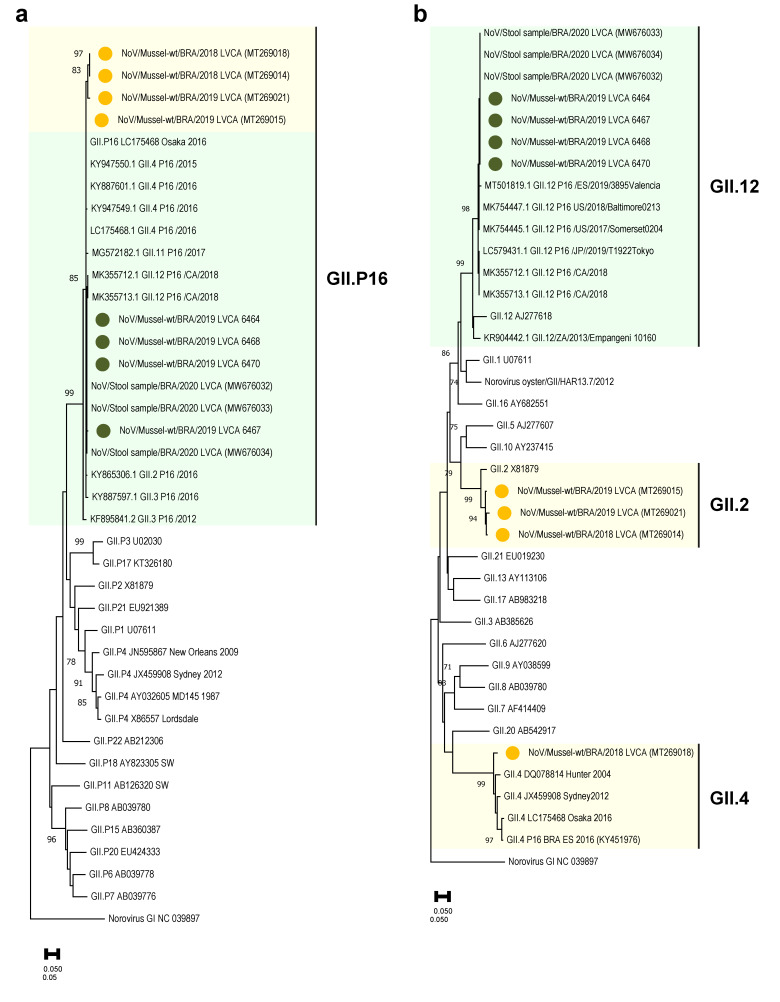
Phylogenetic trees based on (**a**) polymerase and (**b**) capsid regions of GII norovirus. Norovirus GII strains (*n* = 4) isolated from bivalve samples in this study are denoted with a green-filled circle, while GII strains obtained in initial results (*n* = 4) published by Sarmento et al. [17] are marked with a yellow-filled circle. Reference strains were downloaded from the GenBank repository and labelled with their genotype and accession number. Maximum likelihood phylogenetic trees were constructed with MEGA X software with bootstrap tests of 2000 replicates, based on the Kimura two-parameter model. The bootstrap percentage values of ≥65% are shown at each branch point.

Regarding RNA viruses’ characterization, 44.4% (4/9) of norovirus-positive samples collected between August 2019 and February 2020 were successfully characterized and genotyped as GII.12[P16]. GII.12 sequences shared the highest nt sequence identities (*n* = 100%) with Brazilian strains (MW676032, MW676033 and MW676034) detected in clinical samples during 2020, and also showed a high nt identity (*n* = 99%) with G12 strains from other countries, such as Spain (MT501819), EUA (MK754447), Canada (MK355712) and Japan (LC579431). Concerning the polymerase P16 detected, all nt sequences clustered with P16 strains detected in clinical (MW676032, MW676033 and MW676034) and environmental (MT269018 and MT269021) samples from Brazil, showing >99% nt identity (Figure 3).

**Figure 4:**
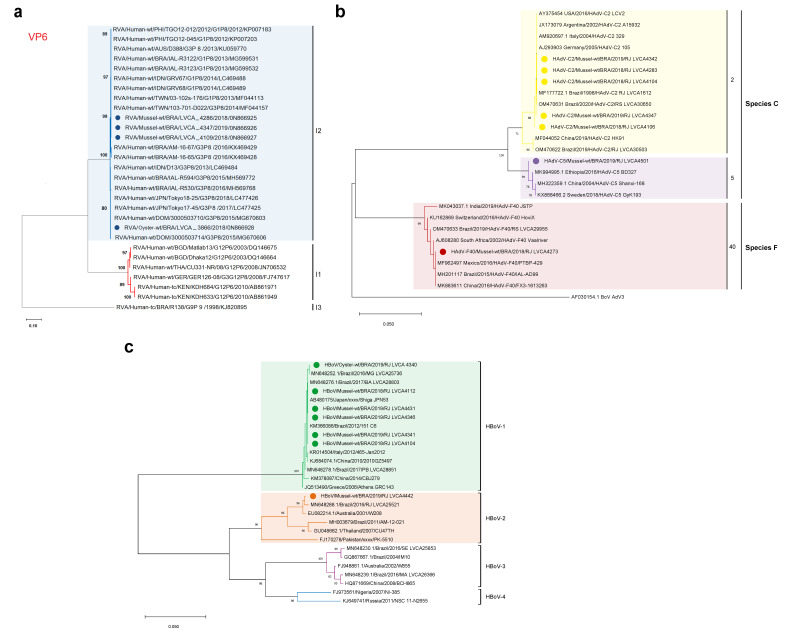
Phylogenetic trees based on (**a**) VP6 nucleotide (nt) of RVA; on (**b**) the *hexon* gene of HAdV; and on (**c**) VP1 nt of HBoV. RVA strains obtained in this study are marked with a blue-filled circle. HAdV-C2, -C5 and -F40 are marked with a yellow, purple and red-filled circle, respectively. HBoV-1 and -2 are marked with a green- and orange-filled circle, respectively. Reference strains were downloaded from GenBank and labelled with their accession number, country of isolation, year, genotype and register number. Phylogenetic trees were generated in MEGA X software with bootstrap tests of 2000 replicates, based on the Tamura 3-parameter model (best fit model for all viruses). Bootstrap values of ≥70% are shown at each branch point.

For RVA, we successfully characterized 25% (4/16) of positive samples, and phylogenetic analysis of the partial VP6 gene characterized sequences as genotype I2. Three bivalve samples detected in 2019 (LVCA_4286, LVCA_4286 and LVCA_4286) shared 100% of nt identity, and one strain from 2018 (LVCA_3866) shared 99% among the others. The three identical sequences showed high nt sequence identities (>99.7%) with G1P[8] strains detected in Indonesia (LC469488 and LC469489), Taiwan (MF044157 and MF044113) and Brazil (KX469429 and KX469428). Whereas the bivalve strain LVCA_3866 was closely related to strains G3P[8] from Japan (LC477425 and LC477424) and Dominican Republic (MG670606), with a nt sequence identity > 99.8% (Figure 4a).

Regarding DNA viruses, we successfully characterized 29.2% (7/24) and 53.8% (7/13) of HAdV- and HBoV-positive samples, respectively. Among the HAdV strains, phylogenetic analysis of a conserved region of the *hexon* gene characterized sequences as belonging to species C (HAdV-2 and -5) and F (HAdV-40), with type C2 as the most frequent (5/7). All HAdV isolates shared 100% nt identity (100%) with strains detected in other countries, with type C2 isolates closely related to strains from Brazil (MF177722 and OM470631), Argentina (JX173079), and Germany (AJ293903 and MH121114). While the type C5 isolated clustered with strains detected in China (MH322359, MH322358 and MH322293), Ethiopia (MK994994 and MK994995) and Sweden (KX868466), and the type F40 isolate clustered with strains from Brazil (MH201117, MT791000 and KY910901), South Africa (MK955316 to MK955319) and Mexico (MF962596 to MF962501) (Figure 4b).

Concerning HBoV, phylogenetic analysis of the partial VP1/VP2 region characterized strains belonging to genotypes HBoV-1 (6/7) and HBoV-2 (1/7). All HBoV-1 strains were genetically related (>99% of nt identity) to previously detected HBoV-1 strains from Brazil (MN648252, MN648276 and KM366086), China (KJ684074), Japan (AB480175) and Italy (KR014504) The detected HBoV-2 strain was closely related (>99%) to Brazilian strains (KX826932, MF034109 and KX826930) and also to strains from Ethiopia (MG383447), Australia (EU082214), United Kingdom (FJ170280) and South Korea (MF680549) (Figure 4c).

## 4. Discussion

In the present study, we investigated the dissemination, viral load and diversity of enteric viruses in bivalve shellfish samples collected in Arraial do Cabo city, between March 2018 and February 2020. Overall, 58.8% of shellfish samples were positive for one or more viruses, while 24.7% tested positive for two or more viruses. Norovirus was the most frequently detected, with the highest viral load among the enteric viruses investigated. No seasonal trend was observed for any of the enteric viruses, in agreement with several previous studies involving clinical, environmental, and wastewater samples from Brazil [41,59,60,61,62,63,64].

Many studies on enteric viruses in shellfish have reported high detection rates for two or more viruses, ranging from 20% to 62%, with norovirus being the most often detected in multiple viral screening in shellfish from Vietnam (81.8%) [65], Singapore (53.3%) [66], Italy (50.2%) [18] and Portugal (37%) [67]. A systematic review of shellfish-borne viral outbreaks has implicated norovirus as the most common viral pathogen associated with shellfish-borne AGE outbreaks [13]. In addition, the most recent report from the European Food Safety Authority identified norovirus as the main etiological agent responsible for AGE outbreaks associated with bivalves consumption [22].

Similar or higher norovirus detection rates were reported in shellfish from the United Kingdom (68.7% and 76.2%) [68,69], India (52.7%) [70] and the Netherlands (45.5%) [20], while studies from the United States, Morocco, South Korea, China, Italy, France and Australia detected lower rates, ranging from <2% to 30% [21,71,72,73,74,75,76,77,78]. A study conducted in a Mangrove Estuary in Vitória city, southeastern Brazil, detected higher norovirus rates (66.7%) in shellfish, followed by RVA (53.3%) and HAdV (46.6%), with 90% of bivalve samples contaminated with at least one enteric viruses [16]. Another Brazilian study, detected norovirus GII in 14% of bivalve samples collected in a Mangrove Estuary from São Paulo state [19].

From August 2019 to February 2020, only norovirus GII was detected, in agreement with the initial data previously published by Sarmento et al. [17], which showed higher rates of GII (87.5%) compared to GI (9.4%). Similarly, studies from India, Italy, Vietnam, Portugal and Spain also found higher norovirus GII detection rates [18,65,67,70,78,79]. The higher detection rate of GII in shellfish samples from Brazil likely reflects the dominance of this genotype circulating among patients with AGE. A recent surveillance study in Brazil, with stool samples from patients with AGE symptoms, demonstrated a high prevalence of norovirus (32.1%), primarily caused by GII (89.1%) [61]. In the same study, GII-infected patients exhibited significantly higher fecal shedding than GI-infected patients (*p* < 0.0001).

We detected higher norovirus concentrations compared to the other enteric viruses. Similar to our findings, norovirus GII concentrations reached 10^7^ GC/g in some bivalve samples in two studies from Southern Italy performed from 2014 to 2017 [18,78]. In China, a one-year study detected norovirus GII at concentrations ranging from 5.0 × 10^3^ to 1.4 × 10^6^ GC/g in oysters collected from seafood markets [75]. In contrast, lower concentrations of norovirus GII were detected in studies from Brazil (5.4 × 10^1^ GC/g) [16], China (1.8 × 10^3^ GC/g) [77] and Vietnam (3.8 × 10^3^ GC/g) [65]. The ability of norovirus to bind to histo-blood group antigens in the digestive tissue of shellfish, therefore increasing their bioaccumulation, is probably linked with the higher levels of norovirus detections seen in this study [80,81].

In our study, HAdV was the second most frequently detected virus. Few studies have reported the presence of HAdV in shellfish, with similar or higher detection rates than ours such as in India, where authors detected HAdV DNA in 21.3% of seafood samples analyzed from retail markets of Mumbai [14]. In Japan, HAdV was found in 52% of the packages of clams [82]. In Brazil, Rigotto et al. [15] detected HAdV in 87.5% of oyster samples, with DNA concentration up to 6 × 10^5^. In Southeastern Brazil, Keller et al. [16] reported DNA concentrations ranging from 4.5 × 10^2^ to 1.2 ×10^3^ GC/g. 

Previous studies in Brazil demonstrated high rates of HAdV contamination of coastal waters, rivers and lagoons [83,84,85,86]. For instance, HAdV was detected in 43% of samples from several beaches along Rio de Janeiro coast, Brazil, and in line with our findings, authors observed a higher detection rate and concentration of specie C compared to specie F [62]. Similarly, a higher prevalence of specie C (26%) than F (1.25%) was also demonstrated in a study with environmental waters, sediment and bivalve samples from Rio Grande do Sul, Brazil [87]. Recently, during a three-year surveillance study in Brazil, our group reported HAdV positivity of 24.5% in stool samples from AGE patients, with higher detection of species F and C [60].

RVA was the third most detected virus, with similar or higher detection rates reported in shellfish from Italy, Argentina, Morocco and Japan, ranging from 12.9% to 57.8% [78,88,89,90]. Compared to our results, other studies detected RVA less frequently in bivalve samples (varying from 0% to 8.3%) in Brazil, Thailand, Italy, South Korea and Singapore [15,18,21,66,74,91]. Regarding RVA concentration, Keller et al. [16] detected viral loads ranging from 2.6 × 10^3^ to 5.3 × 10^3^ GC/g in bivalve samples from Vitória city, Southeastern Brazil. 

The present study is the first evidence of HBoV detection in bivalves in South America. HBoV was the least detected virus, with sequenced samples clustering with HBoV-1 and -2. Worldwide, few studies have investigated the presence of HBoV in bivalves. A one-year study in oysters from Thailand detected HBoV in 7.6% of samples, predominantly genotype 1 [92]. In Italy, two studies identified HBoV in 8.5% and 3.7% of shellfish samples, with detection of genotypes 2 and 3 [21,93]. In South Africa, HBoV was detected in 83% of pooled mussels samples and in 100% of raw sewage, with HBoV-2 and -3 identified in mussel and sewage samples, respectively [94]. Brazil has no data regarding HBoV detection in environmental waters. The nearest geographic report is from Uruguay, where HBoV was detected in 69% and 3% of sewage and surface water samples, respectively, with higher detection rates of HBoV-2 and/or -4 followed by HBoV-3 and -1 [95]. Other countries have also reported high frequencies of HBoV contamination in different water sources, frequently with higher frequencies of genotypes 2 and 3 than genotypes 1 and 4 [96,97,98,99,100].

A recent study in Brazil, detected HBoV in 12.4% of AGE stool samples from children, with higher detection of HBoV-2 and -1 than -3 and fecal shedding up to 1.2 × 10^9^ GC/g [41]. Other studies with hospitalized children for AGE from Northern Brazil also detected HBoV-1, -2 and -3 in stool samples [47,101]. Higher HBoV-1 and -2 detection frequency in stool samples in Brazil may explain their predominance in shellfish in this study. Our results are consistent with those from Thailand, which reported greater detection rates of genotypes 1 and 2, in children and shellfish samples, and genotypes 2 and 1 in sewage samples [46,92,102].

Regarding norovirus characterization, between September 2019 and February 2020, we identified the recombinant strain GII.12[P16]. This recombinant strain was first identified in the United States in 2017 [103] and later detected in Canada, where it was associated with epidemic and endemic AGE, becoming the second most predominant strain during the 2018–2019 epidemic season in that country [104]. Recently, a study in China identified GII.12[P16] in stool and water samples linked to waterborne AGE outbreak [105]. In southern Brazil, this recombinant genotype was detected in stool and ice pop samples related to a foodborne AGE outbreak [9]. Barclay et al. [103] reported the detection of the novel P16 polymerase associated with multiple capsid types, including GII.12, and suggested an increase in viral fitness of recombinant genotypes associated with P16 polymerase.

As for RVA-positive samples, we sequenced the VP6 gene and identified genotype I2, which clustered with human strains of G1P[8] e G3P[8] genotypes isolated in Brazil, Australia and Dominican Republic. Similarly, Marinho et al. [106] identified the genotype I2 from mussels and oysters cultivated in the coastal water of Brazilian Amazon. Recently, the genotype I2 was detected in oyster samples from Argentina, associate with G8-P[1]-I2, which has a bovine-like genome backbone [90]. G3P[8] was the dominant genotype in Brazil in 2018 and 2019, and based on the full genotype constellations, strains were assigned as equine-like G3P[8], and the VP6 gene was determined as genotype I2 in all the stool samples [59,107].

None of the shellfish samples tested positive for HAV. In Brazil, there are few studies regarding HAV detection in naturally contaminated shellfish samples. In Santa Catarina state, southern Brazil, Rigotto et al. [15] did not find any HAV-positive among oysters samples collected. In Rio de Janeiro, HAV was not detected in water or sediment samples [62,108]. Dias et al. [83] detected only one HAV-positive sample out of 48 samples in Rio de Janeiro recreational beach waters. Also in agreement with our findings, no HAV was detected in Australian shellfish [76], and low detection rates were reported in shellfish from the Netherlands (0.2%) [20], South Korea (0.7%) [74], the United States (4.4%) [72], Singapore (8.3%) [66] and Spain (10.1%) [109]. In Italy, Macaluso et al. [110] detected a single positive sample out of the 162 shellfish collected between April 2017 and September 2019 in the Sicily region. Other Italian studies reported HAV detection rates ranging from 8.9% to 16.9% [18,78,111].

The absence of HAV in bivalve samples during our study may be due to improvements in hygienic and sanitary conditions in recent decades in Brazil, including access to safe drinking water. More importantly, the successful inclusion of free HAV vaccination in the National Immunization Program for infants in 2014 led to a sharp decline in the number of registered infected children, the primary affected group [112,113].

Our study has limitations. First, neither the shellfish samples nor the bivalve-growing waters were analyzed for bacteria contamination. Second, physicochemical water parameters and other factors that may affect enteric virus persistence and spread were not assessed. Third, the use of more than one viral surrogate as a procedure control to better assess RNA and DNA virus recoveries was not performed. Fourth, as we used quantitative molecular methodologies and direct sanger sequencing for genotyping, we were unable to differentiate between infectious and non-infectious viral particles and could not explore the viral genetic diversity within the samples. New methodologies based on deep sequencing have recently been developed for foodborne viruses, and although they still face challenges in terms of sensitivity and high costs, their application could allow a better and faster characterization of viral diversity for a more accurate viral surveillance [114].

This study detected pathogenic viral agents in mussels and oysters from Arraial do Cabo, a small touristic coastal city whose population grows by more than 10-fold during the summer. Most shellfish harvests in the region are still predominantly artisanal, and Rio de Janeiro State Environment Institute (INEA) monitors microbiological organisms only at a few beaches for recreational purposes. This is the first identification of the recombinant GII.12[P16] circulating in shellfish, detected around the same time in an outbreak reported in southern Brazil. In addition, the identification of RVA, HBoV, and HAdV strains, previously detected in stool samples from patients with AGE, highlights the problem of high population density, coupled with inadequate urban infrastructure and improperly treated sewage effluents. The regular discharge of untreated sewage into coastal waters negatively affects the marine environment and possibly leads to waterborne or foodborne disease outbreaks. Finally, our study demonstrates the applicability of shellfish biomonitoring as a virological surveillance tool to track and monitor emerging viruses and novel variants.

## Figures and Tables

**Table 1:** Primers and probes used for viral detection and characterization.

Virus	Molecular Method	Primers and Probes	Sequence (5′–3′)	Target Region	Reference
NoV	real-time RT-PCR	COG1F	CGYTGGATGCGNTTYCATGA	ORF1/2 junction	[50]
		COG1R	CTTAGACGCCATCATCATTYAC		
		RING1 (P)	FAM-AGATYGCGATCYCCTGTCCA-TAMRA		
		COG2F	CARGARBCNATGTTYAGRTGGATGAG		
		COG2R	TCGACGCCATCTTCATTCACA		
		RING2 (P)	HEX-TGGGAGGGCGATCGCAATCT-TAMRA		
	RT-PCR	MON431 (F)	TGGACIAGRGGICCYAAYCA	3′ORF1 and 5′ORF2	[51]
		G2SKR (R)	CCRCCNGCATRHCCRTTRTACAT		
RVA	real-time RT-PCR	NSP3-F	ACCATCTWCACRTRACCCTCTATGAG	NSP3	[52]
		NSP3-R1	GGTCACATAACGCCCCTATAGC		
		NSP3 (P)	FAM-AGTTAAAAGCTAACACTGTCAAA-MGB		
	RT-PCR	VP6-F	GACGGVGCRACTACATGGT	VP6 gene	[53]
		VP6-R	GTCCAATTCATNCCTGGTGG		
HVA	real-time RT-PCR	HAV68 (F)	TCACCGCCGTTTGCCTAG	5′-NCR	[54]
		HAV240 (R)	GGAGAGCCCTGGAAGAAAG		
		HAV150p (P)	FAM-TTAATTCCTGCAGGTTCAGG-MGB		
HAdV	real-time PCR	AdF	CATTACATGCACATCGTCCGGG	*Hexon* gene	[55]
		AdR	CAGCGGGCGAAACTTGCACCAG		
		AdP1	FAM-CCGGGCTCAGGTACTCCGAGGCGTCCT-TAMRA		
	PCR	Hex1deg (F)	GCCSCARTGGKCWTACATGCACATC	*Hexon* gene	[56]
		Hex2deg (R)	CAGCACSCCICGRATGTCAAA		
HBoV	real-time PCR	HBoV1 (F)	CCTATATAAGCTGCTGCACTTCCTG	NS1	[57]
		HBoV1 (R)	AAGCCATAGTAGACTCACCACAAG		
		HBoV234 (F)	GCACTTCCGCATYTCGTCAG		
		HBoV3 (R)	GTGGATTGAAAGCCATAATTTGA		
		HBoV24 (R)	AGCAGAAAAGGCCATAGTGTCA		
		(Probe)	FAM-CCAGAGATGTTCACTCGCCG-MGB		
	nested PCR	AK-VP-F1	CGCCGTGGCTCCTGCTCT	VP1/2	[42]
		AK-VP-R1	TGTTCGCCATCACAAAAGATGTG		
		AK-VP-F2	GGCTCCTGCTCTAGGAAATAAAGAG		
		AK-VP-R2	CCTGCTGTTAGGTCGTTGTTGTATGT		

**Table 2:** Single and multiple detections of enteric viruses by bivalve species and collection site.

Bivalve Shellfish	Collecting Site	Samples (*n*)	Number of Positive Samples										
			Single Detection			Double Detection				Triple Detection			Quadruple Detection
			NoV *	RVA	HAdV	NoV + RVA	NoV + HAdV	NoV + HBoV	HBoV + HAdV	NoV + RVA + HAdV	NoV + RVA + HBoV	NoV + HAdV + HBoV	NoV + RVA + HAdV + HBoV
Mussels *(Perna perna)*	Anjos beach	22	3	1	2	0	2	0	0	1	0	2	0
	Farol beach	25	4	1	3	2	4	0	1	0	0	3	0
	Forno beach	26	6	4	0	0	0	2	0	1	0	3	0
Oysters *(Pseudochama cristella)*	Forno beach	24	5	4	0	0	1	0	0	0	1	0	1
Total		97	18	10	5	2	7	2	1	2	1	8	1

* The table displays norovirus results from the 24-month study period, including the initial results published by Sarmento et al. [17] for single and multiple detection comparison. NoV, norovirus; RVA, rotavirus A; HAdV, human adenovirus; HBoV, Human bocavirus.

## Data Availability

The datasets generated and analyzed during the current study are available in the GenBank repository under accession numbers ON815271-ON815277; ON855070-ON855076; ON866925-ON866928 and OP374150-OP374153.

## References

[1] World Health Organization (WHO) (2015). WHO Estimates of the Global Burden of Foodborne Diseases. https://apps.who.int/iris/bitstream/handle/10665/199350/9789241565165_eng.pdf?sequence=1.

[2] Fong T.-T., Lipp E.K. (2005). Enteric Viruses of Humans and Animals in Aquatic Environments: Health Risks, Detection, and Potential Water Quality Assessment Tools. Microbiol. Mol. Biol. Rev..

[3] Gibson K.E. (2014). Viral Pathogens in Water: Occurrence, Public Health Impact, and Available Control Strategies. Curr. Opin. Virol..

[4] Pommepuy M., Dumas F., Caprais M.P., Camus P., Le Mennec C., Parnaudeau S., Haugarreau L., Sarrette B., Vilagines P., Pothier P. (2004). Sewage Impact on Shellfish Microbial Contamination. Water Sci. Technol..

[5] Gerba C.P., Betancourt W.Q., Kitajima M., Rock C.M. (2018). Reducing Uncertainty in Estimating Virus Reduction by Advanced Water Treatment Processes. Water Res..

[6] Kitajima M., Iker B.C., Pepper I.L., Gerba C.P. (2014). Relative Abundance and Treatment Reduction of Viruses during Wastewater Treatment Processes—Identification of Potential Viral Indicators. Sci. Total Environ..

[7] Lanrewaju A.A., Enitan-Folami A.M., Sabiu S., Edokpayi J.N., Swalaha F.M. (2022). Global Public Health Implications of Human Exposure to Viral Contaminated Water. Front. Microbiol..

[8] Fouillet A., Fournet N., Forgeot C., Jones G., Septfons A., Franconeri L., Ambert-Balay K., Schmidt J., Guérin P., de Valk H. (2020). Large Concomitant Outbreaks of Acute Gastroenteritis Emergency Visits in Adults and Food-Borne Events Suspected to Be Linked to Raw Shellfish, France, December 2019 to January 2020. Eurosurveillance.

[9] Fumian T.M., Ferreira F.C., de Andrade J.d.S.R., Canal N., Silva Gomes G., Teixeira L.B., Miagostovich M.P. (2021). Norovirus Foodborne Outbreak Associated With the Consumption of Ice Pop, Southern Brazil, 2020. Food Environ. Virol..

[10] Meghnath K., Hasselback P., McCormick R., Prystajecky N., Taylor M., McIntyre L., Man S., Whitfield Y., Warshawsky B., McKinley M. (2019). Outbreaks of Norovirus and Acute Gastroenteritis Associated with British Columbia Oysters, 2016–2017. Food Environ. Virol..

[11] Le Guyader F.S., Bon F., DeMedici D., Parnaudeau S., Bertone A., Crudeli S., Doyle A., Zidane M., Suffredini E., Kohli E. (2006). Detection of Multiple Noroviruses Associated with an International Gastroenteritis Outbreak Linked to Oyster Consumption. J. Clin. Microbiol..

[12] Bosch A., Gkogka E., Le Guyader F.S., Loisy-Hamon F., Lee A., van Lieshout L., Marthi B., Myrmel M., Sansom A., Schultz A.C. (2018). Foodborne Viruses: Detection, Risk Assessment, and Control Options in Food Processing. Int. J. Food Microbiol..

[13] Bellou M., Kokkinos P., Vantarakis A. (2013). Shellfish-Borne Viral Outbreaks: A Systematic Review. Food Environ. Virol..

[14] Ghosh S.K., Lekshmi M., Das O., Kumar S., Nayak B.B. (2019). Occurrence of Human Enteric Adenoviruses in Fresh Tropical Seafood from Retail Markets and Landing Centers. J. Food Sci..

[15] Rigotto C., Victoria M., Moresco V., Kolesnikovas C.K., Corrêa A.A., Souza D.S.M., Miagostovich M.P., Simões C.M.O., Barardi C.R.M. (2010). Assessment of Adenovirus, Hepatitis A Virus and Rotavirus Presence in Environmental Samples in Florianopolis, South Brazil. J. Appl. Microbiol..

[16] Keller R., Pratte-Santos R., Scarpati K., Martins S.A., Loss S.M., Fumian T.M., Miagostovich M.P., Cassini S.T. (2019). Surveillance of Enteric Viruses and Thermotolerant Coliforms in Surface Water and Bivalves from a Mangrove Estuary in Southeastern Brazil. Food Environ. Virol..

[17] Sarmento S.K., Guerra C.R., Malta F.C., Coutinho R., Miagostovich M.P., Fumian T.M. (2020). Human Norovirus Detection in Bivalve Shellfish in Brazil and Evaluation of Viral Infectivity Using PMA Treatment. Mar. Pollut. Bull..

[18] Fusco G., Anastasio A., Kingsley D.H., Amoroso M.G., Pepe T., Fratamico P.M., Cioffi B., Rossi R., La Rosa G., Boccia F. (2019). Detection of Hepatitis A Virus and Other Enteric Viruses in Shellfish Collected in the Gulf of Naples, Italy. Int. J. Environ. Res. Public Health.

[19] Vasquez-García A., Mejia-Ballesteros J.E., de Godoy S.H.S., Barbieri E., de Sousa R.L.M., Fernandes A.M. (2022). Norovirus GII and Astrovirus in Shellfish from a Mangrove Region in Cananéia, Brazil: Molecular Detection and Characterization. Braz. J. Microbiol..

[20] Dirks R.A.M., Jansen C.C.C., Hägele G., Zwartkruis-Nahuis A.J.T., Tijsma A.S.L., Boxman I.L.A. (2021). Quantitative Levels of Norovirus and Hepatitis A Virus in Bivalve Molluscs Collected along the Food Chain in the Netherlands, 2013–2017. Int. J. Food Microbiol..

[21] Purpari G., Macaluso G., Di Bella S., Gucciardi F., Mira F., Di Marco P., Lastra A., Petersen E., La Rosa G., Guercio A. (2019). Molecular Characterization of Human Enteric Viruses in Food, Water Samples, and Surface Swabs in Sicily. Int. J. Infect. Dis..

[22] European Food Safety Authority, European Centre for Disease Prevention and Control (2021). The European Union One Health 2020 Zoonoses Report. EFSA J..

[23] Burkhardt W., Calci K.R. (2000). Selective Accumulation May Account for Shellfish-Associated Viral Illness. Appl. Environ. Microbiol..

[24] Love D.C., Lovelace G.L., Sobsey M.D. (2010). Removal of Escherichia Coli, Enterococcus Fecalis, Coliphage MS2, Poliovirus, and Hepatitis A Virus from Oysters (Crassostrea Virginica) and Hard Shell Clams (Mercinaria Mercinaria) by Depuration. Int. J. Food Microbiol..

[25] Van Cauwenberghe L., Janssen C.R. (2014). Microplastics in Bivalves Cultured for Human Consumption. Environ. Pollut..

[26] Desdouits M., Piquet J.-C., Wacrenier C., Le Mennec C., Parnaudeau S., Jousse S., Rocq S., Bigault L., Contrant M., Garry P. (2021). Can Shellfish Be Used to Monitor SARS-CoV-2 in the Coastal Environment?. Sci. Total Environ..

[27] Polo D., Lois M., Fernández-Núñez M.T., Romalde J.L. (2021). Detection of SARS-CoV-2 RNA in Bivalve Mollusks and Marine Sediments. Sci. Total Environ..

[28] Mancusi A., Capuano F., Girardi S., Di Maro O., Suffredini E., Di Concilio D., Vassallo L., Cuomo M.C., Tafuro M., Signorelli D. (2022). Detection of SARS-CoV-2 RNA in Bivalve Mollusks by Droplet Digital RT-PCR (Dd RT-PCR). Int. J. Environ. Res. Public Health.

[29] GBD 2015 Mortality and Causes of Death Collaborators (2016). Global, Regional, and National Life Expectancy, All-Cause Mortality, and Cause-Specific Mortality for 249 Causes of Death, 1980–2015: A Systematic Analysis for the Global Burden of Disease Study 2015. Lancet.

[30] Cohen A.L., Platts-Mills J.A., Nakamura T., Operario D.J., Antoni S., Mwenda J.M., Weldegebriel G., Rey-Benito G., Oliveira L.H.d., Ortiz C. (2022). Aetiology and Incidence of Diarrhoea Requiring Hospitalisation in Children under 5 Years of Age in 28 Low-Income and Middle-Income Countries: Findings from the Global Pediatric Diarrhea Surveillance Network. BMJ Global Health.

[31] Chhabra P., de Graaf M., Parra G.I., Chan M.C.-W., Green K., Martella V., Wang Q., White P.A., Katayama K., Vennema H. (2019). Updated Classification of Norovirus Genogroups and Genotypes. J. Gen. Virol..

[32] Greenberg H.B., Estes M.K. (2009). Rotaviruses: From Pathogenesis to Vaccination. Gastroenterology.

[33] Matthijnssens J., Attoui H., Bányai K., Brussaard C.P.D., Danthi P., del Vas M., Dermody T.S., Duncan R., Fāng Q., Johne R. (2022). ICTV Virus Taxonomy Profile: Sedoreoviridae 2022. J. Gen. Virol..

[34] Vaughan G., Goncalves Rossi L.M., Forbi J.C., de Paula V.S., Purdy M.A., Xia G., Khudyakov Y.E. (2014). Hepatitis A Virus: Host Interactions, Molecular Epidemiology and Evolution. Infect. Genet. Evol..

[35] Di Cola G., Fantilli A.C., Pisano M.B., Ré V.E. (2021). Foodborne Transmission of Hepatitis A and Hepatitis E Viruses: A Literature Review. Int. J. Food Microbiol..

[36] Benkő M., Aoki K., Arnberg N., Davison A.J., Echavarría M., Hess M., Jones M.S., Kaján G.L., Kajon A.E., Mittal S.K. (2022). ICTV Virus Taxonomy Profile: Adenoviridae 2022. J. Gen. Virol..

[37] Khanal S., Ghimire P., Dhamoon A.S. (2018). The Repertoire of Adenovirus in Human Disease: The Innocuous to the Deadly. Biomedicines.

[38] Rames E., Roiko A., Stratton H., Macdonald J. (2016). Technical Aspects of Using Human Adenovirus as a Viral Water Quality Indicator. Water Res..

[39] Hundesa A., Maluquer de Motes C., Bofill-Mas S., Albinana-Gimenez N., Girones R. (2006). Identification of Human and Animal Adenoviruses and Polyomaviruses for Determination of Sources of Fecal Contamination in the Environment. Appl. Environ. Microbiol..

[40] Allander T., Tammi M.T., Eriksson M., Bjerkner A., Tiveljung-Lindell A., Andersson B. (2005). Cloning of a Human Parvovirus by Molecular Screening of Respiratory Tract Samples. Proc. Natl. Acad. Sci. USA.

[41] Malta F.C., Varella R.B., Guimarães M.A.A.M., Miagostovich M.P., Fumian T.M. (2020). Human Bocavirus in Brazil: Molecular Epidemiology, Viral Load and Co-Infections. Pathogens.

[42] Kapoor A., Simmonds P., Slikas E., Li L., Bodhidatta L., Sethabutr O., Triki H., Bahri O., Oderinde B.S., Baba M.M. (2010). Human Bocaviruses Are Highly Diverse, Dispersed, Recombination Prone, and Prevalent in Enteric Infections. J. Infect. Dis..

[43] Kapoor A., Slikas E., Simmonds P., Chieochansin T., Naeem A., Shaukat S., Masroor Alam M., Sharif S., Angez M., Zaidi S. (2009). A Newly Identified Bocavirus Species in Human Stool. J. Infect. Dis..

[44] Arthur J.L., Higgins G.D., Davidson G.P., Givney R.C., Ratcliff R.M. (2009). A Novel Bocavirus Associated with Acute Gastroenteritis in Australian Children. PLOS Pathog..

[45] Polo D., Lema A., Gándara E., Romalde J.L. (2022). Prevalence of Human Bocavirus Infections in Europe. A Systematic Review and Meta-Analysis. Transbound. Emerg. Dis..

[46] Nantachit N., Kochjan P., Khamrin P., Kumthip K., Maneekarn N. (2021). Human Bocavirus Genotypes 1, 2, and 3 Circulating in Pediatric Patients with Acute Gastroenteritis in Chiang Mai, Thailand, 2012–2018. J. Infect. Public Health.

[47] Leitão G.A.A., Olivares A.I.O., Pimenta Y.C., Delgado I.F., Miagostovich M.P., Leite J.P.G., de Moraes M.T.B. (2020). Human Bocavirus Genotypes 1 and 2 Detected in Younger Amazonian Children with Acute Gastroenteritis or Respiratory Infections, Respectively. Int. J. Infect. Dis..

[48] (2017). Microbiology of Food a Chain—Horizontal Method for Determination of Hepatitis A Virus and Norovirus in Food Using Real-Time RT-PCR—Part 1: Method for Quantification.

[49] Rajal V.B., McSwain B.S., Thompson D.E., Leutenegger C.M., Kildare B.J., Wuertz S. (2007). Validation of Hollow Fiber Ultrafiltration and Real-Time PCR Using Bacteriophage PP7 as Surrogate for the Quantification of Viruses from Water Samples. Water Res..

[50] Kageyama T., Kojima S., Shinohara M., Uchida K., Fukushi S., Hoshino F.B., Takeda N., Katayama K. (2003). Broadly Reactive and Highly Sensitive Assay for Norwalk-like Viruses Based on Real-Time Quantitative Reverse Transcription-PCR. J. Clin. Microbiol..

[51] Kojima S., Kageyama T., Fukushi S., Hoshino F.B., Shinohara M., Uchida K., Natori K., Takeda N., Katayama K. (2002). Genogroup-Specific PCR Primers for Detection of Norwalk-like Viruses. J. Virol. Methods.

[52] Zeng S.-Q., Halkosalo A., Salminen M., Szakal E.D., Puustinen L., Vesikari T. (2008). One-Step Quantitative RT-PCR for the Detection of Rotavirus in Acute Gastroenteritis. J. Virol. Methods.

[53] Iturriza Gómara M., Wong C., Blome S., Desselberger U., Gray J. (2002). Molecular Characterization of VP6 Genes of Human Rotavirus Isolates: Correlation of Genogroups with Subgroups and Evidence of Independent Segregation. J. Virol..

[54] Costafreda M.I., Bosch A., Pintó R.M. (2006). Development, Evaluation, and Standardization of a Real-Time TaqMan Reverse Transcription-PCR Assay for Quantification of Hepatitis A Virus in Clinical and Shellfish Samples. Appl. Environ. Microbiol..

[55] Hernroth B.E., Conden-Hansson A.-C., Rehnstam-Holm A.-S., Girones R., Allard A.K. (2002). Environmental Factors Influencing Human Viral Pathogens and Their Potential Indicator Organisms in the Blue Mussel, Mytilus Edulis: The First Scandinavian Report. Appl. Environ. Microbiol..

[56] Allard A., Albinsson B., Wadell G. (2001). Rapid Typing of Human Adenoviruses by a General PCR Combined with Restriction Endonuclease Analysis. J. Clin. Microbiol..

[57] Kantola K., Sadeghi M., Antikainen J., Kirveskari J., Delwart E., Hedman K., Söderlund-Venermo M. (2010). Real-Time Quantitative PCR Detection of Four Human Bocaviruses. J. Clin. Microbiol..

[58] Kumar S., Stecher G., Li M., Knyaz C., Tamura K. (2018). MEGA X: Molecular Evolutionary Genetics Analysis across Computing Platforms. Mol. Biol. Evol..

[59] Gutierrez M.B., Fialho A.M., Maranhão A.G., Malta F.C., Andrade J.d.S.R.d., Assis R.M.S.d., Miagostovich M.P., Leite J.P.G., Machado Fumian T. (2020). Rotavirus A in Brazil: Molecular Epidemiology and Surveillance during 2018–2019. Pathogens.

[60] Nascimento L.G., Fialho A.M., Andrade J.d.S.R., Assis R.M.S., Fumian T.M. (2022). Human Enteric Adenovirus F40/41 as a Major Cause of Acute Gastroenteritis in Children in Brazil, 2018 to 2020. Sci. Rep..

[61] Sarmento S.K., de Andrade J.d.S.R., Miagostovich M.P., Fumian T.M. (2021). Virological and Epidemiological Features of Norovirus Infections in Brazil, 2017–2018. Viruses.

[62] Staggemeier R., Heck T.M.S., Demoliner M., Ritzel R.G.F., Röhnelt N.M.S., Girardi V., Venker C.A., Spilki F.R. (2017). Enteric Viruses and Adenovirus Diversity in Waters from 2016 Olympic Venues. Sci. Total Environ..

[63] Assis A.S.F., Fumian T.M., Miagostovich M.P., Drumond B.P., da Rosa E., Silva M.L. (2018). Adenovirus and Rotavirus Recovery from a Treated Effluent through an Optimized Skimmed-Milk Flocculation Method. Environ. Sci. Pollut. Res. Int..

[64] Fumian T.M., Fioretti J.M., Lun J.H., Dos Santos I.A.L., White P.A., Miagostovich M.P. (2019). Detection of Norovirus Epidemic Genotypes in Raw Sewage Using next Generation Sequencing. Environ. Int..

[65] Suffredini E., Le Q.H., Di Pasquale S., Pham T.D., Vicenza T., Losardo M., To K.A., De Medici D. (2020). Occurrence and Molecular Characterization of Enteric Viruses in Bivalve Shellfish Marketed in Vietnam. Food Control.

[66] Tan M.T.H., Ho S.X., Chu J.J.H., Li D. (2021). Application of Virome Capture Sequencing in Shellfish Sold at Retail Level in Singapore. Lett. Appl. Microbiol..

[67] Mesquita J.R., Vaz L., Cerqueira S., Castilho F., Santos R., Monteiro S., Manso C.F., Romalde J.L., Nascimento M.S.J. (2011). Norovirus, Hepatitis A Virus and Enterovirus Presence in Shellfish from High Quality Harvesting Areas in Portugal. Food Microbiol..

[68] Lowther J.A., Gustar N.E., Powell A.L., Hartnell R.E., Lees D.N. (2012). Two-Year Systematic Study to Assess Norovirus Contamination in Oysters from Commercial Harvesting Areas in the United Kingdom. Appl. Environ. Microbiol..

[69] Lowther J.A., Gustar N.E., Powell A.L., O’Brien S., Lees D.N. (2018). A One-Year Survey of Norovirus in UK Oysters Collected at the Point of Sale. Food Environ. Virol..

[70] Das O., Lekshmi M., Kumar S., Nayak B.B. (2020). Incidence of Norovirus in Tropical Seafood Harbouring Fecal Indicator Bacteria. Mar. Pollut. Bull..

[71] Benabbes L., Ollivier J., Schaeffer J., Parnaudeau S., Rhaissi H., Nourlil J., Le Guyader F.S. (2013). Norovirus and Other Human Enteric Viruses in Moroccan Shellfish. Food Environ. Virol..

[72] DePaola A., Jones J.L., Woods J., Burkhardt W., Calci K.R., Krantz J.A., Bowers J.C., Kasturi K., Byars R.H., Jacobs E. (2010). Bacterial and Viral Pathogens in Live Oysters: 2007 United States Market Survey. Appl. Environ. Microbiol..

[73] Schaeffer J., Le Saux J.-C., Lora M., Atmar R.L., Le Guyader F.S. (2013). Norovirus Contamination on French Marketed Oysters. Int. J. Food Microbiol..

[74] Seo D.J., Lee M.H., Son N.R., Seo S., Lee K.B., Wang X., Choi C. (2014). Seasonal and Regional Prevalence of Norovirus, Hepatitis A Virus, Hepatitis E Virus, and Rotavirus in Shellfish Harvested from South Korea. Food Control.

[75] Tao J., Chunhui H., Fanning S., Nan L., Jiahui W., Hongyuan Z., Jing Z., Fengqin L. (2018). Norovirus Contamination in Retail Oysters from Beijing and Qingdao, China. Food Control.

[76] Torok V., Hodgson K., McLeod C., Tan J., Malhi N., Turnbull A. (2018). National Survey of Foodborne Viruses in Australian Oysters at Production. Food Microbiol..

[77] Zhang H., Liu D., Zhang Z., Hewitt J., Li X., Hou P., Wang D., Wu Q. (2021). Surveillance of Human Norovirus in Oysters Collected from Production Area in Shandong Province, China during 2017–2018. Food Control.

[78] Fusco G., Di Bartolo I., Cioffi B., Ianiro G., Palermo P., Monini M., Amoroso M.G. (2017). Prevalence of Foodborne Viruses in Mussels in Southern Italy. Food Environ. Virol..

[79] Vilariño M.L., Le Guyader F.S., Polo D., Schaeffer J., Kröl J., Romalde J.L. (2009). Assessment of Human Enteric Viruses in Cultured and Wild Bivalve Molluscs. Int. Microbiol..

[80] Le Guyader F.S., Atmar R.L., Le Pendu J. (2012). Transmission of Viruses through Shellfish: When Specific Ligands Come into Play. Curr. Opin. Virol..

[81] Maalouf H., Zakhour M., Le Pendu J., Le Saux J.-C., Atmar R.L., Le Guyader F.S. (2010). Distribution in Tissue and Seasonal Variation of Norovirus Genogroup I and II Ligands in Oysters. Appl. Environ. Microbiol..

[82] Hansman G.S., Oka T., Li T.-C., Nishio O., Noda M., Takeda N. (2008). Detection of Human Enteric Viruses in Japanese Clams. J. Food Prot..

[83] Dias J., Pinto R.N., Vieira C.B., de Abreu Corrêa A. (2018). Detection and Quantification of Human Adenovirus (HAdV), JC Polyomavirus (JCPyV) and Hepatitis A Virus (HAV) in Recreational Waters of Niterói, Rio de Janeiro, Brazil. Mar. Pollut. Bull..

[84] Miagostovich M.P., Ferreira F.F.M., Guimarães F.R., Fumian T.M., Diniz-Mendes L., Luz S.L.B., Silva L.A., Leite J.P.G. (2008). Molecular Detection and Characterization of Gastroenteritis Viruses Occurring Naturally in the Stream Waters of Manaus, Central Amazonia, Brazil. Appl. Environ. Microbiol..

[85] Pedrosa de Macena L.D.G., Castiglia Feitosa R., Vieira C.B., Araújo I.T., Taniuchi M., Miagostovich M.P. (2021). Microbiological Assessment of an Urban Lagoon System in the Coastal Zone of Rio de Janeiro, Brazil. Environ Sci. Pollut. Res. Int..

[86] Victoria M., Fumian T.M., Rocha M.S., Dalmao F., Leite J.P.G., Girones R., Miagostovich M.P. (2014). Gastroenteric Virus Dissemination and Influence of Rainfall Events in Urban Beaches in Brazil. J. Appl. Microbiol..

[87] Gularte J.S., Girardi V., Demoliner M., de Souza F.G., Filippi M., Eisen A.K.A., Mena K.D., de Quevedo D.M., Rigotto C., de Barros M.P. (2019). Human Mastadenovirus in Water, Sediment, Sea Surface Microlayer, and Bivalve Mollusk from Southern Brazilian Beaches. Mar. Pollut. Bull..

[88] Hatib A., Hassou N., Benani A., Hafid J.E., Ennaji M.M. (2021). Molecular Detection of Rotavirus in Mollusks from the Oued El Maleh Estuary of Mohammedia, Morocco. J. Pure Appl. Microbiol..

[89] Ito E., Pu J., Miura T., Kazama S., Nishiyama M., Ito H., Konta Y., Nguyen G.T., Omura T., Watanabe T. (2019). Weekly Variation of Rotavirus A Concentrations in Sewage and Oysters in Japan, 2014–2016. Pathogens.

[90] Mozgovoj M., Miño S., Barbieri E.S., Tort F.L., Victoria-Montero M., Frydman C., Cap M., Baron P.J., Colina R., Matthijnssens J. (2022). GII.4 Human Norovirus and G8P[1] Bovine-like Rotavirus in Oysters (Crassostrea Gigas) from Argentina. Int. J. Food Microbiol..

[91] Kittigul L., Panjangampatthana A., Rupprom K., Pombubpa K. (2014). Genetic Diversity of Rotavirus Strains Circulating in Environmental Water and Bivalve Shellfish in Thailand. Int. J. Environ. Res. Public Health.

[92] Kumthip K., Khamrin P., Ushijima H., Maneekarn N. (2021). Predominance of Human Bocavirus Genotypes 1 and 2 in Oysters in Thailand. Appl. Environ. Microbiol..

[93] La Rosa G., Purpari G., Guercio A., Di Bella S., Gucciardi F., Proroga Y.T.R., Pisanu M., Della Libera S., Iaconelli M., Suffredini E. (2018). Detection of Human Bocavirus Species 2 and 3 in Bivalve Shellfish in Italy. Appl. Environ. Microbiol..

[94] Onosi O., Upfold N.S., Jukes M.D., Luke G.A., Knox C. (2020). The First Detection of Human Bocavirus Species 2 and 3 in Raw Sewage and Mussels in South Africa. Food Environ. Virol..

[95] Salvo M., Lizasoain A., Castells M., Bortagaray V., Castro S., Colina R., Tort F.L., Victoria M. (2018). Human Bocavirus: Detection, Quantification and Molecular Characterization in Sewage and Surface Waters in Uruguay. Food Environ. Virol..

[96] Blinkova O., Rosario K., Li L., Kapoor A., Slikas B., Bernardin F., Breitbart M., Delwart E. (2009). Frequent Detection of Highly Diverse Variants of Cardiovirus, Cosavirus, Bocavirus, and Circovirus in Sewage Samples Collected in the United States. J. Clin. Microbiol..

[97] Hamza H., Leifels M., Wilhelm M., Hamza I.A. (2017). Relative Abundance of Human Bocaviruses in Urban Sewage in Greater Cairo, Egypt. Food Environ. Virol..

[98] Hamza I.A., Jurzik L., Wilhelm M., Überla K. (2009). Detection and Quantification of Human Bocavirus in River Water. J. Gen. Virol..

[99] Iaconelli M., Divizia M., Della Libera S., Di Bonito P., La Rosa G. (2016). Frequent Detection and Genetic Diversity of Human Bocavirus in Urban Sewage Samples. Food Environ. Virol..

[100] La Rosa G., Sanseverino I., Della Libera S., Iaconelli M., Ferrero V.E.V., Barra Caracciolo A., Lettieri T. (2017). The Impact of Anthropogenic Pressure on the Virological Quality of Water from the Tiber River, Italy. Lett. Appl. Microbiol..

[101] Soares L.S., Lima A.B.F., Pantoja K.C., Lobo P.S., Cruz J.F., Guerra S.F.S., Bezerra D.A.M., Bandeira R.S., Mascarenhas J.D.P. (2019). Molecular Epidemiology of Human Bocavirus in Children with Acute Gastroenteritis from North Region of Brazil. J. Med. Microbiol..

[102] Kumthip K., Khamrin P., Yodmeeklin A., Ushijima H., Maneekarn N. (2021). Contamination of Human Bocavirus Genotypes 1, 2, 3, and 4 in Environmental Waters in Thailand. Microbiol. Spectr..

[103] Barclay L., Cannon J.L., Wikswo M.E., Phillips A.R., Browne H., Montmayeur A.M., Tatusov R.L., Burke R.M., Hall A.J., Vinjé J. (2019). Emerging Novel GII.P16 Noroviruses Associated with Multiple Capsid Genotypes. Viruses.

[104] Pabbaraju K., Wong A.A., Tipples G.A., Pang X.-L. (2019). Emergence of a Novel Recombinant Norovirus GII.P16-GII.12 Strain Causing Gastroenteritis, Alberta, Canada. Emerg. Infect. Dis..

[105] Xiong Q., Jiang H., Liu Z., Peng J., Sun J., Fang L., Li C., Qiu M., Zhang X., Lu J. (2022). Untangling an AGS Outbreak Caused by the Recombinant GII.12[P16] Norovirus With Nanopore Sequencing. Front. Cell. Infect. Microbiol..

[106] Marinho A.N.R., Rocha D.C.C., Kanai Y.K., Alves C.M., Costa D.C., Sousa A.H., Barros B.C.V., Bonfim M.C.M.S., Mascarenhas J.D.P. (2018). Rotavirus Analyses by SYBR Green Real-Time PCR and Microbiological Contamination in Bivalves Cultivated in Coastal Water of Amazonian Brazil. J. Water Health.

[107] Gutierrez M.B., de Figueiredo M.R., Fialho A.M., Cantelli C.P., Miagostovich M.P., Fumian T.M. (2021). Nosocomial Acute Gastroenteritis Outbreak Caused by an Equine-like G3P[8] DS-1-like Rotavirus and GII.4 Sydney[P16] Norovirus at a Pediatric Hospital in Rio de Janeiro, Brazil, 2019. Hum. Vaccin. Immunother..

[108] Miagostovich M.P., Guimarães F.R., Vieira C.B., Fumian T.M., da Gama N.P., Victoria M., de Oliveira J.M., de Oliveira Mendes A.C., Gaspar A.M.C., Leite J.P.G. (2014). Assessment of Water Quality in a Border Region between the Atlantic Forest and an Urbanised Area in Rio de Janeiro, Brazil. Food Environ. Virol..

[109] Polo D., Varela M.F., Romalde J.L. (2015). Detection and Quantification of Hepatitis A Virus and Norovirus in Spanish Authorized Shellfish Harvesting Areas. Int. J. Food Microbiol..

[110] Macaluso G., Guercio A., Gucciardi F., Di Bella S., La Rosa G., Suffredini E., Randazzo W., Purpari G. (2021). Occurrence of Human Enteric Viruses in Shellfish along the Production and Distribution Chain in Sicily, Italy. Foods.

[111] La Rosa G., Mancini P., Bonanno Ferraro G., Iaconelli M., Veneri C., Paradiso R., De Medici D., Vicenza T., Proroga Y.T.R., Di Maro O. (2020). Hepatitis A Virus Strains Circulating in the Campania Region (2015–2018) Assessed through Bivalve Biomonitoring and Environmental Surveillance. Viruses.

[112] De Oliveira T.M., Vieira N.S.G., Sepp T.D.S., Souto F.J.D. (2020). Recent Trends in Hepatitis A Incidence in Brazil. J. Med. Virol..

[113] Souto F.J.D., de Brito W.I., Fontes C.J.F. (2019). Impact of the Single-Dose Universal Mass Vaccination Strategy against Hepatitis A in Brazil. Vaccine.

[114] Desdouits M., de Graaf M., Strubbia S., Oude Munnink B.B., Kroneman A., Le Guyader F.S., Koopmans M.P.G. (2020). Novel Opportunities for NGS-Based One Health Surveillance of Foodborne Viruses. One Health Outlook.

